# A Rare but Important Clinical Presentation of Induced Methemoglobinemia

**DOI:** 10.5811/westjem.2016.6.30504

**Published:** 2016-07-19

**Authors:** Faried Banimahd, Tricia Loo, Manish Amin, Omeed R. Ahadiat, Bharath Chakravarthy, Shahram Lotfipour

**Affiliations:** * University of California, Irvine, Department of Emergency Medicine, Irvine, California; † University of California, San Francisco, Department of Emergency Medicine, Fresno, California; ‡ University of California, Los Angeles, Department of Emergency Medicine, Kern Medical Center, Bakersfield, California; § Rosalind Franklin University of Medicine and Science, Chicago Medical School, North Chicago, Illinois

## INTRODUCTION

Phenazopyridine is considered one of the classic causes of drug-induced methemoglobinemia. It is often taught as such and seen in board review courses. Nevertheless, the epidemiology is unknown, presentation quite rare, and less than five cases have been reported in PubMed in over 35 years.^1^–^4^ We present a case with a different set of patient characteristics than seen in the few recent case reports, and an approach to treatment that validates further uniqueness, justifying reporting the case in the literature. In particular, the patient was a young otherwise-healthy adult, with the initial diagnosis and decision to treat based on clinical grounds versus laboratory values.

## CASE REPORT

A 32-year-old otherwise-healthy female presented to the emergency department (ED) with a chief complaint of lower abdominal pain, dysuria, urinary frequency, and hesitation for four days. On the day of presentation she started to have worsening symptoms and left flank pain.

Her temperature was 36.7 Celsius, heart rate of 87 beats per minute, blood pressure of 144/84, respiratory rate of 24 breaths per minute, and pulse oximetry of 85% on room air. In the resuscitation bay she was placed on monitor, and oxygen was delivered via non-rebreather 10-liter facemask, with an oxygen saturation of 79% (Image 1).

On initial evaluation she denied shortness of breath, cough, congestion, chest pain, or any other respiratory symptoms. She was alert and oriented, but stated that something did not feel right. Upon further questioning she reported taking phenazopyridine three times daily for the preceding three days. At one point during the interview, her oxygen saturation dropped to the mid 60s, but the patient did not report any symptoms. The rest of her history was non-contributory except for the occasional use of nicotine and a childhood episode of hospitalization for a kidney infection. Upon physical examination, she appeared to be pale but in no apparent respiratory distress. She also had a slight bluish/gray discoloration on her lips (Image 2).

Breath sounds were clear bilaterally. She had left costo-vertebral angle tenderness, and was tender in the suprapubic area of the abdomen, without rebound or guarding. At this point, it was determined that she most likely had methemoglobinemia secondary to phenazopyridine ingestion, in addition to pyelonephritis. Methylene blue was ordered from the pharmacy and we proceeded with the laboratory testing, and other diagnostic evaluations.

Laboratory tests noted hemoglobin of 10.3 g/dL, and hematocrit of 31.4%. Urinalysis was dark brown, hazy, positive for nitrates, large leukocyte esterase, many bacteria, and >180 WBCs/HPF. The methemoglobin level was 11.9%. The blood from this draw was noted to be chocolate brown (Image 3).

Prior to the return of the ordered laboratory studies, methylene blue was received. We felt that our history and physical exam gave us a very strong clinical suspicion for the diagnosis of methemoglobinemia and pyelonephritis. Given our concern for the prolonged central cyanosis in conjunction with the comorbidity of the pyelonephritis, we decided to proceed and treat the methemoglobinemia. She was given methylene blue 100mg IV. Shortly after receiving the methylene blue, she stated that she felt that her mind had “cleared up,” and that she felt less “foggy.” Her oxygen saturation level improved to the low 90s on room air.

At this time, the internal medicine (IM) service was consulted for admission and she was started on ceftriaxone 1g intravenous for her pyelonephritis. A second methemoglobin level was obtained by the IM service six hours after the dose of methylene blue and it was 0%. Approximately 24 hours after admission, oxygen saturation was documented at 99% on room air, with an arterial blood gas (ABG) reading of pH=7.5/pCO2=28/pO2=123/HCO3=21/BE=0.

The patient was in the hospital for three days. Her hospital course was unremarkable.

Prior to discharge, she no longer had urinary symptoms, and she felt markedly improved. Her discharge instructions included “do not ever use phenazopyridine!”

## DISCUSSION

Phenazopyridine is a commonly used over-the-counter (OTC) medication reported by patients presenting to the ED with complaints of dysuria. Conversely, methemoglobinemia induced from phenazopyridine ingestion is rarely seen in the ED. The epidemiology is unknown and very few cases are cited in the literature, with most in the remote past.^1^–^4^ Cases of methemoglobinemia are most frequently cited in the literature after use of dapsone and local anesthetics (i.e. benzocaine and lidocaine), and rarely reported in the emergency medicine literature.^4^–^7^

Methemoglobin forms when the ferrous (Fe2+) irons of heme are oxidized to the ferric (Fe3+) state. In the ferric state, methemoglobin is unable to bind oxygen. In addition, the oxygen affinity of any remaining ferrous heme in the hemoglobin tetramer is increased. As a result, the oxygen dissociation curve is shifted to the left, resulting in hypoxia and lactic acid production.^8^

Methemoglobin is reduced to ferrous hemoglobin by two pathways. The main pathway—which is the only physiologically important pathway—is the NADH-dependent reaction catalyzed by cytochrome b5 reductase (b5R). The alternative pathway—which is not physiologically active—uses NADPH generated by glucose-6-phosphate dehydrogenase (G6PD) in the hexose monophosphate shunt to reduce methemoglobin to hemoglobin. Extrinsically administered electron acceptors, such as methylene blue, are required for this pathway to be activated.^9^

Methemoglobinemia can be congenital or acquired.^9^ Congenital causes are seen in cytochrome b5 reductase deficiency or hemoglobin M disease. All patients with hereditary methemoglobinemia should avoid exposure to aniline derivatives, nitrates, and other agents that may, even in normal individuals, induce methemoglobinemia.

Induced or acquired causes occur when methemoglobin production is accelerated beyond the capacity of NADH reductase activity. This usually occurs as a drug reaction.

Methemoglobinemia may be clinically suspected by the presence of clinical “cyanosis” in the presence of a normal arterial pO2 (PaO2) obtained by arterial blood gases. The blood in methemoglobinemia has been variously described as dark red, chocolate, or brownish to blue in color, and, unlike deoxy-hemoglobin, the color does not change with the addition of oxygen (Image 3).

In asymptomatic patients, usually those with methemoglobin levels <20 percent, no therapy other than discontinuation of the offending agent(s) suffices. Patients with acutely acquired methemoglobinemia may be asymptomatic at lower levels of methemoglobin (i.e., <20 percent). Methylene blue therapy is indicated if the patient is symptomatic, or if the methemoglobin level is >20 percent. Symptoms, when present, include headache, fatigue, dyspnea, and lethargy. At methemoglobin levels >40 percent, respiratory depression, altered consciousness, shock, seizures, and death may occur. Blood transfusion or exchange transfusion may be helpful in patients who are in shock. Hyperbaric oxygen has been used with anecdotal success in severe cases.

Methylene blue given intravenously in a dose of 1 to 2 mg/kg over five minutes provides an artificial electron transporter for the ultimate reduction of methemoglobin via the NADPH-dependent pathway. The response is usually rapid; the dose may be repeated in one hour if the level of methemoglobin is still high one hour after the initial infusion, but retreatment is frequently not necessary.^10^

In our patient, the decision to treat was based on the history and physical exam in addition to the urinalysis. Our trigger was the fact that the co-morbidity of the kidney infection and its potential anaerobic stress as well as the consideration of possibly extensive lab-result lag time in a busy county trauma center.

Although rarely seen, it is important to remember that this commonly used and accessible medication can precipitate methemoglobinemia. This case illustrates multiple classic teaching modules in emergency medicine including central cyanosis and dyshemoglobinemia. This case also illustrates the invaluable utility of a good history. This can be done rapidly and with directive, even in a busy modern ED.

## Figures and Tables

**Figure 1:**
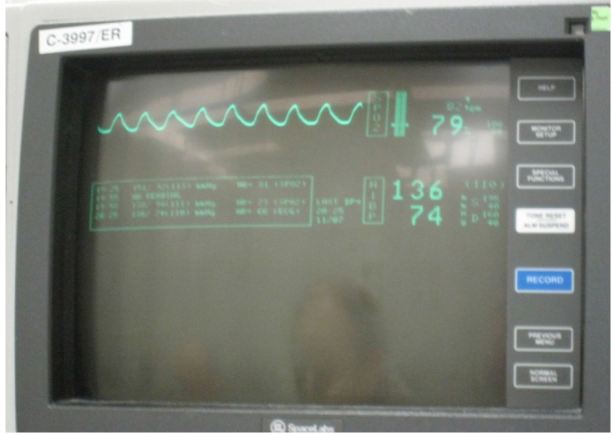
Monitor displays oxygen saturation of 79%.

**Figure 2:**
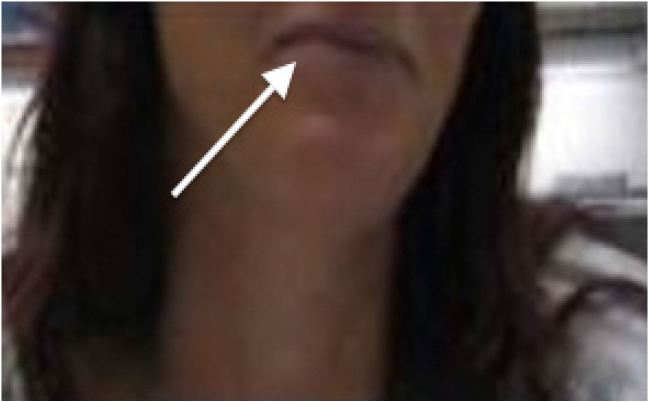
White arrow indicates blue/grey discoloration of lips**.**

**Figure 3:**
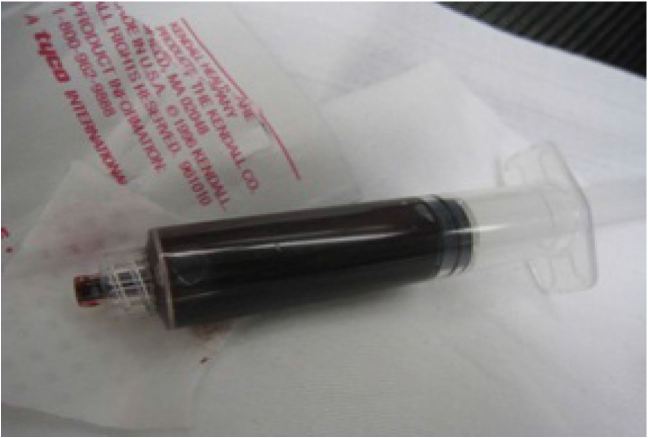
Blood grossly appears chocolate brown color.
